# Identifying characteristics and outcomes that are associated with fall-related fatalities: multi-year retrospective summary of fall deaths in older adults from 2005–2012

**DOI:** 10.1186/s40621-017-0117-8

**Published:** 2017-07-24

**Authors:** Sara M. Deprey, Lynda Biedrzycki, Kristine Klenz

**Affiliations:** 1 Carroll University, 100 N East Ave, Waukesha, WI 53186 USA; 2 Waukesha County Medical Examiner’s Office, Waukesha, 53188 USA

**Keywords:** Elderly, Accidental falls, Fatal outcome

## Abstract

**Background:**

Fall-related deaths continue to be the leading cause of accidental deaths in the older adult (65+ year) population. However, many fall-related fatalities are unspecified and little is known about the fall characteristics and personal demographics at the time of the fall. Therefore, this report describes the characteristics, circumstances and injuries of falls that resulted in older adult deaths in one U.S. County and explores the variables associated with fatal injuries from falls.

**Methods:**

This is a continued retrospective analysis of 841older adults whose underlying cause of death was due to a fall over an 8-year period (2005–2012). Demographics and logistic regression of fall characteristics and injuries were analyzed.

**Results:**

Falls that led to death most often occurred when walking in one’s own home. Most of the residents in this study were community-dwellers who had previous comorbidities taking an average of six medications prior to their fall. Survival after a fall was on average 31 days. The two most common injuries after a fatal fall were hip fractures (54%), and head injuries (21%). A logistic regression identified two variables associated with hip fracture, advancing age (OR = 1.05, 95% confidence interval [CI] = 1.02–1.08) and diagnosis of a prior neurological condition (OR = 2.1, 95% CI = 1.4–3.1). Variables associated with head injuries included younger age (OR = .91, 95% CI = .89–.94), male gender (OR = 2.5, 95% CI = 1.7–3.8), prescribed anticoagulants (OR = 2.4, 95% CI = 1.5–3.9) and negative musculoskeletal comorbidity (OR = 1.9. 95% CI = 1.1–3.0).

**Conclusion:**

Hip fractures and head injuries were the most common injury after a fall that led to death in older adults greater than 65 years. There are opposing risk factors for older adults who incur a hip fracture compared to a head injury. Thus, health professionals will need to individualize prevention efforts to reduce fall fatalities.

## Background

There has been considerable attention and resources directed towards prevention of falls and fall-related deaths over the past 20 years (Province et al. 1995; Bell et al. 2000; Rubenstein et al. 2002; Stevens et al. 2015). However, there are consistent reports that 25–33% of adults over the age of 65 years continue to fall each year (Shumway-Cook et al. 2009; Milat et al. 2011; Bergen et al. 2014). Though some studies report serious injuries such as fractures or head injuries in less than 3% of all falls, these fall injuries are the most common causes of mortality from falls (Stevens et al. 2014; Deprey 2009). Fall-related deaths continue to be the leading cause of accidental deaths in the older adult (65+ year) population (CDC 2016). Although it has been suggested that recent reporting of fall-related deaths is more inconclusive and may better reflect the actual sequel of falls, the rate of overall fall fatalities continues to increase (Gagne et al. 2013). Several studies have analyzed national vital statistics to identify fall-related death trends (Gagne et al. 2013; Alamgir et al. 2012; Stevens and Rudd 2014). These studies have allowed analysis of personal variables such as age, gender, race and circumstances surrounding falls that are listed in National databases. Trends suggest increasing mortality rates due to falls in older adults with mortality rates highest with advancing age and among whites. Fall deaths from stairs, steps and ladders have been decreasing, yet falls from activities on the same level have increased (Gagne et al. 2013; Alamgir et al. 2012; Stevens and Rudd 2014). However, using vital statistics data does not give specific information about the personal and environmental factors related to the actual fall, as a fall may have occurred several weeks or months preceding death (Thierauf et al. 2010). Authors have found that statistics that rely on death certificate reviews underreport accidentals deaths due to falls (Koehler et al. 2006). Using individual medical records taken at the time of the fall along with investigator reports may help to augment death certificate information and provide additional information about the personal and environmental factors at the time of a fall that eventually resulted in death. In addition, when using CDC WONDER online database (CDC 2016) unspecified falls, though decreasing, made up 49.1% of all US fall fatalities in 2010, thus the circumstances of nearly half of all fall-related deaths are unknown (CDC 2016). Using information that was documented at the time of the fall may lead to better understanding of fall deaths.

Therefore, the purposes of this study are to provide a descriptive analysis of the personal characteristic, circumstances and injuries surrounding a fall that eventually resulted in older adult fatality in one United States (U.S.) County and explore the characteristics associated with fall injuries that led to fatalities.

## Methods

### Study design and participants

This retrospective study is a continuation of a pilot study (Deprey 2009) and part of a larger multi-year summary of older adult fall fatalities in Waukesha county, Wisconsin from 2005 to 2012 (Deprey et al. 2015). Data from all County community-dwelling and institutionalized persons ≥65 years whose underlying cause of death was due to an injury incurred during a fall were reviewed. A fall was determined to be the underlying cause of death if, after the medical examiner’s investigation, the fall injury “initiated the chain of events that led directly and inevitably to death” ((U.S. Department of Health and Human Services. 2003) pg. 14). Data from hospital and nursing facility medical records, medical examiner’s investigation and examination records, and documented interviews with significant others (health practitioners, paramedics, family members, caregivers, and/or others involved in the safety of the faller) were collected at the County’s medical examiner’s office for the purposes of this study. Ethical consent was granted from the author’s institution to carry out and disseminate data collection (IRB #13-016).

After a fall, older adults may acquire additional medical diagnoses (e.g., pneumonia) or require additional medication that are associated with their death. However, the aim of this study’s data collection was to describe a person’s medical status and circumstances at the time of their fall. Variables that have been previously associated with older adult falls were collected for analysis (Tinetti and Ginter 1988; Nevitt et al. 1991; Tinetti et al. 1995).

### Personal characteristics

Demographics collected for the study included, age, gender, and body mass index (BMI) at the time of death. The individual’s place of residence when the fall occurred was grouped into community, institution, or assisted living. Individuals who lived in their own home or apartment were considered community-dwellers. Institution-dwellers were people who were living in a skilled nursing facility or were in a hospital or hospice at the time of their fall. Assisted living was kept as a separate category due to the variety of services offered at assisted living facilities. Survival time after the fall was calculated as the number of days from the fall to death.

To gain a picture of an individual’s medical status, comorbidities documented in the records at the time of the fall, not at the time of death, were also included in this study. Comorbidities were grouped into systems of Cardiovascular (e.g., hypertension, atrial fibrillation, heart failure), Neurological (e.g., Parkinson’s disease, stroke, dementia) and Musculoskeletal (e.g., previous joint replacement, fractures) (Tinetti and Ginter 1988; Nevitt et al. 1991; Tinetti et al. 1995; Rubenstein 2006). Specific diagnostic subgroups were also collected. Hypertension was categorized as a subgroup of cardiovascular disease as there has been an association with falls and hypertension (Tinetti et al. 2014). Osteoporosis, including a diagnosis of osteopenia, was collected and categorized separately from musculoskeletal conditions as it may have an association with hip fractures, a common injury after a fall leading to death (Melton et al. 1986; Cummings et al. 1993). Osteoarthritis was chosen as an additional variable due its potential role in gait deviations leading to falls (Nevitt et al. 1989; Hausdorff et al. 1997). Number of prescribed medications (not including vitamins) at the time of the fall were investigated as multiple medications are a risk factor for falls (Leipzig et al. 1999). Anticoagulant medications have been associated with head injuries after falls (Chisholm and Harruff 2010). Therefore, prescribed anticoagulant medications documented in the medical or investigation records were dichotomously categorized. We included anticoagulants as well as all antiplatelet medication such as clopidgrel or aspirin as someone who was taking anticoagulants at the time of their fall. This is consistent with how other authors categorized anticoagulants (Brewer et al. 2011).

### Fall circumstances and injuries

Location of the fall, activity that the individual was participating in when they fell, and the injury incurred from the fall were chosen as additional variables to analyze for this study. Location of the fall was listed as a specific room within the home or residence of the individual, outside of the home (e.g., yard, driveway, or garage) or community which included areas such as, but not limited to parking lots, restaurants, and offices. Activity the individual was engaged in at the time of the fall was considered a ground level activity or an activity occurring on a stair, step or ladder. Ground level activities were categorized into walking, slip or trip, transfers (e.g., sit to stand or moving from bed to chair), falling from furniture (e.g., bed or couch), picking an object up from the ground, or standing task (e.g., washing dishes). Falls were considered ground level unless the investigation records stated the person fell from a ladder or different level such as a roof and the fall was stated to be from a height of more than one meter. Falls on stairs or steps were categorized separately from ground level. Medical injuries resulting from the fall were gathered and listed by the specific injury reported in the investigation or medical records. Cranial subdural hematomas, epidurals or intracranial hemorrhage, were all grouped as head injuries. Skull fractures were listed separately from head injuries and recorded as skull fractures. Unspecified category was used if circumstances of the fall were unknown or the variable or circumstance of the fall was so specific it may have revealed the identity of the resident.

### Statistical analysis

In addition to frequencies and descriptive statistics to summarize the data, we looked at differences between gender, age and primary injuries from falls. Age was used as a continuous variable as well as stratified into three groups of 65–74 years, 75–84 years and 85+ years. To identify differences among continuous variables, an independent *t*-test or one way analysis of variance (ANOVA) was used. Chi Square was used to analyze proportions between categorical variables. Once frequent injuries or conditions suffered in fatal falls were identified, a binary logistic regression was used to assess which independent variables may explain the likelihood of incurring specific injuries. Injury or medical condition were considered the dependent variable in the logistic regression model. SPSS (Version 24.0. Armonk, NY: IBM Corp.) and Microsoft Excel 2010 were used for descriptive statistics and graphing. Alpha level of <0.05 was considered statistically significant.

When analyzing data, we excluded cases pairwise, i.e., we excluded the resident only if they were missing the data required for the specific analysis (Pallant 2007). Additionally, we excluded cases from specific analyses if the outcome was greater than four standard deviations from the mean.

## Results

A total of 842 fall-related deaths were identified in Waukesha county from 2005–2012. One subject whose fall was 18 years prior to their death was excluded from the analysis as detailed information about the fall could not be determined. Therefore, records of 841 people were analyzed for this report. County population from the 2010 census representative of this 8 year study period was 389,891 in which 14% of the population is 65+ years. Females comprised 56.8% of the over 65-year population. However, the proportion of females over 85 years was 68.4% in 2010 (U.S. Census Bureau: State and County QuickFacts 2014).

**Fig. 1:**
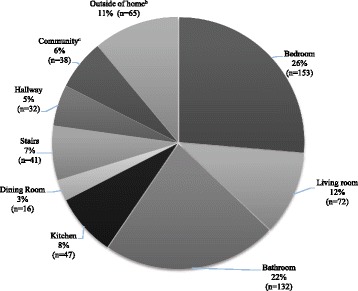
Fall location that led to fatality, Waukesha County, Wisconsin (*N* = 596). ^b^ Outside of home included driveway, yard, garage. ^c^ Community included parking lot, resturant, store, curb step etc

**Fig. 2:**
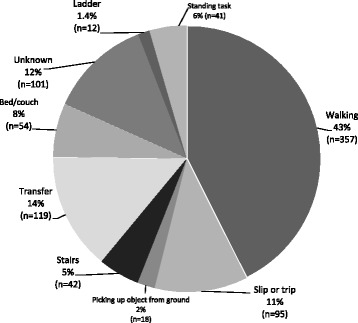
Fall Activity: Activity at the time of a fall that led to a fatality in Waukesha County, Wisconsin (*N* = 839)

The personal and clinical characteristic of the residents from this study are shown in Table 1. Less than 20% of all falls occurred outside of the home or in the community, with majority of the falls occurring at home. Please refer to Fig. 1. A significantly higher proportion of men fell outside the home (34.7%) compared to women (15.4%), whereas more women (79.4%) than men (57.1%) fell inside the home, (x^2^ = 34.696, *p* = 0.00, effect size Phi 0.243). Ground level activities, such as walking, caused the most falls that resulted in fatality. Please refer to Fig. 2.

**Table 1:** Demographics of older adult fall-related fatalities, Waukesha County, Wisconsin 2005–2012

	All (*n* = 841)	65–74 years (*n* = 61)	75–84 years (*n* = 273)	85+ years (*n* = 507)
Age mean (SD)	86.0 (7.23)	70.8 (2.75)	80.6 (2.48)	90.8 (3.98)
Females	61.2%	47.5%	53.8%	66.9%
BMI^a^				
mean (SD)	23.5 (5.83)	26.4 (7.94)	24.7 (6.30)	22.5 (4.94)*
Residence: (*n* = 834)				
Home or apartment (n)	55.2% (464)	80% (49)	63.4% (173)	47.7% (242)
Assisted living (n)	22.6% (190)	4.9% (3)	16.1% (44)	28.2% (143)
Institution (n)	21.4% (180)	14.4% (9)	20.1% (55)	22.9% (116)
Survival time^b^				
Number of days from fall to death mean (SD)	31.2 (44.78)	25.9 (42.23)	31.8 (50.84)	31.4 (41.57)
Comorbidities^c^				
Cardiovascular (*n* = 806)	87% (*n* = 701)	78.6% (44)	87.4% (228)	87.7% (429)
Hypertension (*n* = 796)	77.4% (*n* = 616)	77.4% (41)	78% (202)	77% (373)
Neurological (*n* = 804)	57.3% (*n* = 461)	41.1%* (23)	56.8% (147)	59.5% (291)
Musculoskeletal (*n* = 783)	32.1% (*n* = 253)	30.4% (17)	33.5% (85)	31.6% (151)
Osteoporosis (*n* = 792)	23.5% (*n* = 186)	10.7%* (6)	21.6% (55)	26% (125)
Osteoarthritis (*n* = 157)	27.2% (*n* = 157)	18.6%* (8)	20.9% (37)	31.4% (112)
Number of prescribed medications mean (SD)	6.23 (3.6)	6.3 (4.27)	6.85 (3.86)*	5.89 (3.32)*
Anticoagulants prescribed^c^ (*n* = 745)	56% (*n=*417)	51.9% (27)	56.8% (137)	56% (253)
Fall Location: (*n* = 596)				
At home^d^ (n)	82.7% (493)	55.7% (34)	60. 4% (165)	58% (294)
Outside of home^e^ (n)	10.9% (65)	14.8% (9)	9.9% (27)	5.7% (29)
Community^f^ (n)	6.4% (38)	4.9% (3)	5.9% (16)	3.7% (19)
Fall Injury: (*n* = 834)				
Hip fracture (n)	54.6% (456)	35.6% (21)	47.8% (130)	60.5% (305)*
Head injury (n)	21.6% (180)	45% (27)*	27.5% (75)	15.4% (78)

*Statistically significant differences (*p* < 0.05)

^a^BMI = Body mass index (kg/m^2^)

^b^two cases were excluded as the mean was > 4 SD from the mean

^c^ proportion of positive findings from available data

^d^ Primary residence

^e^Outside of home included driveway, yard, garage

^f^Community included parking lot, store, restaurant, curb step etc

**Fig. 3:**
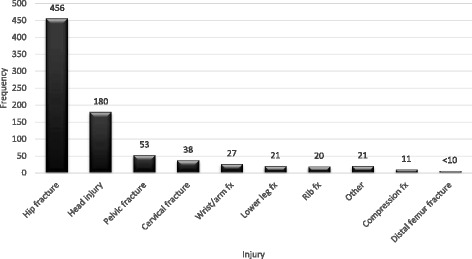
Injuries Incurred from Falls Contributing Death in Waukesha County, Wisconsin (*N* = 834)

Hip fractures and head injuries were the two most common injuries that occurred after a fall that led to death (See Fig. 3). While there are “other” injuries such as contusions, chest trauma or abdominal injuries that occurred from 2.4% of the county’s fall fatalities, most of the fall outcomes involved skeletal fractures or head injuries.

Univariate analyses were performed to identify personal characteristics that may be statistically different between hip fractures and head injuries. The youngest age group had proportionally more head injuries than the other age groups, that is 45% of all injuries in the 65–74 year group were head injuries compared to the 27.5% in the 75–84 and 15.4% in the >85 year olds (x^2^ = 36.56 *p* = 0.00, effect size Cramer’s V = .21). In contrast, the oldest age group (>85 years) had proportionally more hip fractures (60.5%) compared to the other age groups (x^2^ = 20.80 *p* = 0.00, effect size Cramer’s V = 1.6). Table 2 displays the difference in age, gender and BMI that resulted in a fatal head injury or hip fracture after a fall.

**Table 2:** Age, gender BMI, and survival differences of persons after a fatal head injury or hip fracture, Waukesha County, Wisconsin 2005-2012

	Head Injury (*n* = 180)	Hip Fractures (*n* = 456)
Age (SD)*	82.81 y (7.72)	87.23 y (6.76)
Males	57.78% (*n* = 104)	34.21% (*n* = 156)
Females	42.22% (*n* = 76)	65.78% (*n* = 300)
BMI^a^ (SD)*	24.72 kg/m^2^ (4.98) (*n* = 159)	22.53 kg/m^2^ (5.64) (*n* = 386)
Survival time* # Days fall to death^b^	20.56 (35.1) ^b^	35.96 (50.5)

*Statistically significant differences (*p* < 0.05)

*y* years

^a^BMI = body mass index (kg/m^2^)

^b^two cases were excluded as the mean was > 4 SD from the mean

We identified that 417 (56%) residents who suffered a fall fatality were prescribed anticoagulants. In our study population, those who were prescribed anticoagulant medications were statistically more likely to die from a head injury due to their fall than those not prescribed anticoagulants (x^2^ = 9.849, *p* = 0.002, effect size Phi = .16). In contrast, the residents in this study who suffered a fall fatality and not prescribed anticoagulants were statistically more likely to suffer a hip fracture (x^2^ = 4.272, *p* = 0.045, effect size small Phi = 0.076).

Logistic regression was used to further explore the relationship between variables that may explain the likelihood of incurring a fatal hip fracture or head injury after a fall. The first logistic regression was used to determine which variables were associated with hip fracture. Eight independent variables were added to the model that were statistically significant in the prior analyses or associated with hip fractures (Tinetti and Ginter 1988; Nevitt et al. 1991; Tinetti et al. 1995; Rubenstein 2006; Tinetti et al. 2014; Melton et al. 1986; Cummings et al. 1993; Cauley et al. 2000). Variables included, age, gender, BMI, number of medications, cardiovascular disease, neurological disease, osteoarthritis, and osteoporosis. The full model containing the eight aforementioned predictors was statistically significant, X^2^ (8, *N* = 400) =40.13, *p* = .000, suggesting the model was able to identify persons whose cause of death was due to a hip fracture. The model as a whole explained 12.7% (Nagelkerke pseudo R^2^) of the variance in hip fractures and correctly classified 62% of the cases. As shown in Table 3, age and diagnosis of a neurological condition were the only two statistically significant contributions to the model.

**Table 3:** Logistic Regression: Variables associated with fatal hip fractures after a fall, Waukesha County, Wisconsin 2005–2012

Variables included in Model	B	Std Error	Wald	df	p*	Odds Ratio	95% Confidence Interval	
Age*	.05	.02	8.67	1	.003	1.05	1.02	1.08
Gender^a^	.34	.23	2.21	1	.14	1.40	.90	2.20
BMI^b^	−.04	.02	2.95	1	.09	.97	.93	1.01
Number of medications	.04	.03	1.61	1	.205	1.04	.98	1.11
Cardiovascular Comorbidity	−.33	.34	9.33	1	.33	.72	.37	1.40
Neurological* Comorbidity	.72	.22	11.26	1	.001	2.06	1.35	3.14
Osteoarthritis	.23	.24	.88	1	.35	1.26	.78	2.02
Osteoporosis	.21	.27	.60	1	.44	1.23	.73	2.07
Constant	−3.79	1.52	6.27	1	.012	.02		

*Statistically significant (*p* < 0.05)

^a^males coded = 0 females coded = 1

^b^BMI = body mass index (kg/m^2^)

A second logistic regression was performed to explain the variables that may be associated with death due to head injury after a fall. This model contained eight independent variables that were associated with head injuries, age, gender, BMI, number of medications, prescribed anticoagulant medications, cardiovascular disease, neurological disease, and musculoskeletal conditions (Chisholm and Harruff 2010). The full model containing all predictors was statistically significant, X^2^ (8, *N* = 583) =91.27, *p* = .000, suggesting the model was able to identify persons whose cause of death was due to a head injury. The model as a whole explained 21.8% (Nagelkerke pseudo R^2^) of the variance in head injuries and correctly classified 79.4% of the cases. Table 4 displays the statistically significant contributions to the model.

**Table 4:** Logistic Regression: Variables associated with fatal head injuries after a fall, Waukesha County, Wisconsin 2005–2012

Variables included in Model	B	Std Error	Wald	df	p*	Odds Ratio	95% Confidence Interval	
Age (years)*	−.09	.012	32.73	1	.00	.91	.89	.94
Gender^a^*	−.92	.22	18.24	1	.00	2.5	1.65	3.83
BMI^b^	.01	.02	.36	1	.55	1.01	.97	1.05
Number of medications	−0.48	.032	2.25	1	.13	.95	.90	1.02
Anticoagulant medication prescribed*	.88	.24	13.04	1	.00	2.4	1.5	3.88
Cardiovascular comorbidity	−.14	.32	.181	1	.67	.87	.46	1.64
Neurological comorbidity	−.33	.22	2.29	1	.13	.72	.472	1.10
Musculoskeletal comorbidity*	−.63	.25	6.42	1	.01	1.87	1.15	3.05
Constant	6.9	1.50	21	1	.00	1012.71		

*Statistically significant (*p* < 0.05)

^a^males coded = 0 females coded = 1

^b^BMI = body mass index (kg/m^2^)

## Discussion

The purpose of this report was to describe the common characteristics, circumstances and injuries after a fall that led to the death of 841 older adults in one U.S. County over an 8 year period. In addition, we explored the personal characteristics of specific fall injuries leading to fatalities. Falls that led to death most often occurred when walking in one’s own home. Most of the residents in this study were community-dwellers who had previous comorbidities. Hip fractures (54%), and head injuries (21%) were the two most common injuries after a fall. A logistic regression identified two variables, advancing age and diagnosis of a prior neurological condition associated with hip fractures. Whereas, variables associated with head injuries included male gender, younger age, prescribed anticoagulants and negative musculoskeletal condition. Despite the injury, survival after the fall was on average 31 days, suggesting a significant decline in health between a fall and the month following injury.

Residents were more likely to experience their fall and resultant injury inside their home. Ground level activities, mainly walking, was the primary activity engaged in when a fatal fall occurred, this is consistent with other non-fatal and fatal fall reports (Milat et al. 2011; Stevens et al. 2014). This might suggest unfamiliarity of one’s surrounding is an unlikely cause of falls that led to fatal injuries. However, we could not establish if a person was dependent in their home environment, had gait instability, required assistance or needed a device when walking, all which have been associated with falls (Tinetti and Ginter 1988; Rubenstein et al. 1994; Studenski et al. 2011). Thus, a more likely explanation for the number of fatal falls in one’s home was the combination of comorbidities and medications in this study population that may have contributed to a pre-fall decline in daily functional abilities. Previous studies have suggested that multiple comorbidities and multiple medication use has been associated with decreased mobility and gait instability, both linked to falls (Stevens and Rudd 2014; Rubenstein 2006; Tinetti et al. 2014; Hausdorff et al. 1997; Leipzig et al. 1999). Greater than 85% of the people in this study had a comorbidity and were taking an average of six prescribed medications therefore potentially increasing their fall risk and poor post-fall functional recovery (Gill et al. 2013).

A common place for an injurious fall leading to mortality within the home was the bedroom and bathroom. This is consistent with others that investigated injurious and fatal falls (Stevens et al. 2014; Stevens and Rudd 2014; Chisholm and Harruff 2010). Thus, we agree with the recommendations from Stevens et al (Stevens et al. 2014) that current fall prevention efforts and resources should be focused on bathroom safety including financial assistance (e.g., insurance coverage) for bathroom items such as installation of walk in showers, tubs, non-slip flooring and at the very least secure hand rails and bath seats. Falls on stairs and ladders occurred in 6.4% of the study population. This is similar to U.S. trends in the same time period (Stevens and Rudd 2014). The low occurrence of falls from stairs or ladders may indicate more safety measures are in place on stairs or more education has been provided about the dangers of falls from heights (Centers for Disease Control and Prevention, National Center for Injury Prevention and Control, Division of Unintentional Injury Prevention 2016).

A small percentage of fall fatalities occurred outside of the home and community. This could suggest that older adults who fall and die perhaps have less access to the community. There is limited public transportation in this particular county, hence if one does not drive or is unable to drive, access to the community limits fall opportunities in that environment. On the other hand, less community falls may suggest that persons may select to limit outdoor or community participation.

### Injuries

Skeletal fractures (76%) and head injuries (21%) accounted for most of the injuries that led to mortality after a fall. Less than three percent of the injuries were due to contusion, chest, or abdominal injuries. Thus, falls resulting in soft tissue injuries did not initiate the fatal sequel of medical or functional decline as much as skeletal fractures or head injuries did. Hip fractures and head injuries were the two most frequent injuries after a fall that led to a fatality. The youngest age group, 65–74 years, had proportionally more head injuries and were mostly men. Most, 80%, of the younger age group lived in their own home suggesting perhaps a greater amount of functional independence and participation in activities that increased the risk of head injuries.

### Hip fracture

To further explore the relationship of personal characteristics in this study, we used a logistic regression to identify which variables were associated with falls resulting in fatal hip fractures. We included variables in the regression model that have been previously correlated with falls or hip fractures (Tinetti and Ginter 1988; Nevitt et al. 1991; Tinetti et al. 1995; Melton et al. 1986; Leipzig et al. 1999). Though we had more women in our study, gender was not a predictor of hip fracture in our model. Perhaps this might be due to the majority (68%) of the County's 85+ population is female. Previous reports have found as age increases, injury rates after falls increase in both men and women (Stevens et al. 1999; Samelson et al. 2002). Though age was found to be statistically significant within our model, an odds ratio of 1.05 seems to suggest that age is a contributor of hip fracture but the risk did not increase as substantially as previously reported risks (Samelson et al. 2002; Scott 1990). Thus, there seems to be more variability in health and function as individuals age that may be more associated with mortality, than age alone (Magaziner et al. 1997; Shumway-Cook et al. 2005). The regression model did however find two times greater risk of mortality after a hip fracture in older adults with a prior fall neurological comorbidity than an older adult without a neurological disorder. Our category of neurological comorbidity included people with Parkinson’s disease and stroke each having a reported fall risk two times greater than the general population (Wood et al. 2002; Jorgensen et al. 2002). Dementia was also categorized as a neurological comorbidity in this study and has been linked to an eight fold increase in fall risk (Allan et al. 2009). Additionally, consequences of many neurological disorders result in functional and gait limitations which have been proposed as fall risk factors (Hausdorff et al. 1997). Many of the persons in the older age group did not live in their own home suggesting that functional abilities were more compromised requiring daily institutional assistance. Therefore, ameliorating functional deficits after neurological injury may reduce fall risk, but this would require further investigation.

Though others have predicted low bone density increases the likelihood of fractures, a low BMI or diagnosis of osteoporosis was not predictive of hip fracture in our model (Cummings et al. 1993; Cauley et al. 2000). We collected data on both BMI and reported diagnosis of osteoporosis. To make sure the two were not directly related we tested for multicollinearity and found they were not related in this population. We believed collecting BMI data may have demonstrated a more accurate picture of bone mineral density than a diagnosis of osteoporosis or osteopenia that could have been underreported (Salamat et al. 2013). The average BMI for persons suffering a hip fracture in this study was 22.5 kg/m^2^. This has been reported to increase the likelihood of osteoporotic fractures (De Laet et al. 2005). However, for consistency, we collected BMI at time of death as many medical records did not record height or weight at the time of injury. This may not have been reflective of what a person’s BMI was the time of their fall, especially if the fall was on average 36 days prior to the declining medical condition and resultant death.

Overall 87% of the variance associated with hip fracture in this study were not explained by the variables in the model. This may indicate that additional factors other than age and comorbidities may be responsible for outcomes after hip fracture such as functional mobility before and after a fall (Gill et al. 2013; Eastwood et al. 2002). Follow up procedures such as continued rehabilitation to reduce possible gait deviations and maximize functional potential, may reduce mortality given death often occurs approximately 1 month after a hip fracture, which is after one is discharged from the hospital (Shumway-Cook et al. 2005; Studenski 2013). Prospective studies are recommended to investigate older adult fallers who enter the medical system with a hip fracture to monitor their course of medical and functional recovery over a 6 month period to further elucidate the variables for morbidity and mortality.

### Head injuries

Head injuries occurred in 180 residents over the 8 year study period accounting for 21.6% of all injuries. Four variables in our regression model increased the likelihood of a head injury in our study sample, male gender, younger age ($\overline{\mathrm{x}}$ = 82.9 years versus 86 years for all other injuries), prescribed anticoagulants and lack of pre-fall musculoskeletal conditions.

Significant variables associated with a fatal head injury from a fall are not consistent with the average demographics of this study. Significant variables of age, gender and prescribed anticoagulants are consistent with the findings of Chisholm et al. (Chisholm and Harruff 2010), who analyzed ground level falls that resulted in mortality in older adults (Chisholm and Harruff 2010). Both studies found men were at higher risk for head injuries. The likelihood of incurring a head injury was 2.5 times greater for men in this study than women. The average age of people who incurred a head injury was 82.9 years in this study and 82 years in Chisholm’s study (Chisholm and Harruff 2010). The negative regression coefficient for age suggests younger age in our sample was associated with head injuries, again similar to what Chisholm et al (Chisholm and Harruff 2010) found in their study population. Both studies found a higher likelihood of incurring a head injury when residents were taking anticoagulants, 2.4 times in the present study and 4.67 times more likely in Chisholm et al (Chisholm and Harruff 2010). Several authors (Brewer et al. 2011; Howard et al. 2009) found that patients taking anticoagulants or antiplatelet medications have a higher risk of intracranial hemorrhage after a mild head trauma (mean age 79 years) with (Brewer et al. 2011) or without (Howard et al. 2009) a loss of consciousness. Often, anticoagulated older adults do not present with typical signs and symptoms of head injury, that is, they presented with a higher Glasgow Coma Score, presented to the emergency department awake and thus were often under triaged (Brewer et al. 2011; Howard et al. 2009; Ivascu et al. 2005). These studies suggest that quicker triage, implementation of head CT scan even when loss of consciousness is not present and point of care INR should be done to identify those asymptomatic older adults after a ground level fall. The higher risk of head injuries after a fall in older adults who are prescribed anticoagulation medication may provide awareness of the potentially fatal consequences in the absence of typical signs and symptoms.

The fourth variable in our logistic regression model found predictive of fatal head injuries not identified in in other studies, was the lack of a musculoskeletal condition prior to a fall. There was 1.8 times the risk of a fatal head injury after a fall in persons who did not have previous musculoskeletal disorders. This is difficult to interpret, but in combination with the negative correlations with other comorbidities in the regression model suggests that perhaps this group of adults were healthier or functionally more mobile than the rest of the study population. Thus, those who incurred a head injury may present with very different risk factors than 76% of people in this study who incurred a skeletal fracture. Perhaps future studies should investigate fatal head injuries independently from other fatal falls and include younger age ranges to identify potential risk factors.

There are opposing risk factors for older adults who incur a head injury compared to a hip fracture. The variables associated with falls that resulted in fatal head injuries such as prescribed anticoagulants can provide some insight into prevention. However, variables associated with falls resulting in hip fractures and consequently fatalities, remain elusive. The statistically longer survival time and lower BMI of people incurring a hip fracture are different than persons who suffered a head injury. Thus, the variables that predispose a person to a hip fracture after a fall may be more related to pre-fall and post-fall health status (Gill et al. 2013; Shumway-Cook et al. 2005). Thus, efforts to prevent fall fatalities may need to target different risk factors depending on the health status of the individual.

### Limitations

There were several limitations in this study. First and foremost, due to the retrospective nature of this data there is no assumption of cause and effect in this study. Secondly, data concerning a person’s prior functional status were inconsistent. Consequently, we categorized where a person was residing when they fell making the assumption that a community-dweller would be functionally more independent than an institutional-dweller. There are however, inherent limitations in this assumption given that most falls occurred at one’s home. Another limitation of this report is the potential for under reporting the impact of comorbidities. Hence, we don’t know if risk increases in a person who has multiple system comorbidities. Lastly, we included prescribed medications in our analyses. If there was a medication effect it is unknown if the medication itself, or non compliance with the medication, was associated with fall fatalities.

## Conclusions

Hip fractures and head injuries were the most common injury after a fall that led to death in persons 65 years or older. There are opposing risk factors for mortality in older adults who incur a hip fracture compared to a head injury. Older age and prior neurological condition are significant variables associated with death after a fall resulting in a hip fracture. Where as younger males prescribed anticoagulants were more likely to die after a fall resulting in a head injury. Thus, health professionals will need to individualize prevention efforts to reduce fall fatalities.
